# Unveiling the influence of persuasion strategies on cognitive engagement: an ERPs study on attentional search

**DOI:** 10.3389/fnbeh.2024.1302770

**Published:** 2024-09-10

**Authors:** Lichao Xiu, Xuejiao Chen, Lulu Mao, Enyu Zhang, Guoming Yu

**Affiliations:** Laboratory of Cognitive Neuroscience and Communication, School of Journalism and Communication, Beijing Normal University, Beijing, China

**Keywords:** elaboration likelihood model, attention, ERP, visual search, persuasion

## Abstract

The objective of this study was to explore the impact of different persuasive strategies, as delineated in the Elaboration Likelihood Model (ELM), on attentional processes using event-related potentials (ERPs).

**Introduction:**

This study aimed to investigate how central versus peripheral persuasion methods, delivered through rational and emotional persuasion strategies, influence cognitive engagement and information processing during visual search tasks.

**Methods:**

Participants were allocated into four groups based on the media type (video vs. text) and the persuasion route (central vs. peripheral). The early and late stages of attentional processing were examined through the N1, P2, and P3 ERP components.

**Results:**

The results demonstrated a pronounced N1 amplitude in response to text-based peripheral persuasion, indicating enhanced early attentional engagement. Additionally, parallel search tasks revealed a larger P3 amplitude for central versus peripheral routes, suggesting significant cognitive resource allocation during tasks requiring higher attention.

**Discussion:**

These findings underscore the nuanced role of persuasive strategies in modulating attentional resources and cognitive processing. The study offers insights into designing more effective communication messages and highlights the potential for tailored persuasion approaches to influence audience engagement and information processing, with implications for public health campaigns and beyond.

## Introduction

The paramount instrument for terminating the COVID-19 pandemic resides in vaccination; however, pervasive misinformation surrounding the pandemic engenders vaccine hesitancy among populations. This hesitancy manifests through individuals questioning the efficacy of vaccines, expressing concerns regarding their safety, doubting the necessity for vaccination, and often associating vaccines with certain diseases (Dror et al., 2020; Lazarus et al., 2020; Palamenghi et al., 2020; Hernandez et al., 2021). Consequently, the rates of vaccination have not reached the desired levels. By the conclusion of March 2022, in excess of 11 billion doses of the COVID-19 vaccine had been disseminated, yet approximately 36 percent of the global populace had not received the initial dose, as reported by Reuters (Tencent News, 2022). The predominant challenge at present, therefore, entails persuading individuals to partake in vaccination.

### The elaboration likelihood model

Prior research emphasizing the persuasive impact of vaccines predominantly explored vaccination intentions or attitudes towards vaccines (Head et al., 2020; Kim et al., 2020; Lazarus et al., 2020; Loomba et al., 2021; Chen et al., 2022). Additionally, there exists a body of work investigating vaccine-related misinformation and the subsequent countermeasures and interventions to mitigate such misinformation (Islam et al., 2021; Kim et al., 2021; Scannell et al., 2021). A significant portion of these studies are anchored in the Elaboration Likelihood Model (ELM), which adeptly elucidates the persuasion process and validates the efficacy of both the central and peripheral routes to persuasion. Within this model, attention and comprehension are posited as fundamental prerequisites (Petty and Cacioppo, 1986).

The Elaboration Likelihood Model (ELM) serves as a foundational framework for understanding the mechanisms of persuasion (Manca et al., 2019). This model delineates two primary routes of persuasion: the central and the peripheral. The central route is characterized by a high level of cognitive engagement, where persuasion is contingent on the strength and logic of the arguments presented (Cacioppo and Petty, 1982). Conversely, the peripheral route relies on superficial cues such as the attractiveness or credibility of the source, rather than the argument’s content (Petty and Cacioppo, 1986). Understanding these routes is crucial for comprehending how individuals process persuasive communications, a key aspect explored in this study.

In recent years, certain studies have advanced the Elaboration Likelihood Model (ELM), accentuating the role of mediating variables such as concentration and cognitive resources (Harrington et al., 2006; Zha et al., 2018). These studies scrutinize the extent to which individuals engage in thoughtful processing of persuasive messages, as well as their selection of persuasion routes. However, in the so-called “post-truth era,” wherein emotion supersedes fact and attitude overrides cognition in communication dynamics (Xiu et al., 2020), individuals often exhibit a diminished attention span toward information provided by sources. Consequently, traditional persuasion research, which centers on behavioral intention and attitude change, appears to have diverged from contemporary reality.

### Attention and persuasion strategy

It is acknowledged that attitudes are resilient to change (Kelly and Barker, 2016; Winter et al., 2021), hence, a shift in focus toward the cognitive and emotional facets, as opposed to solely behavioral intentions, is advocated. More importantly, we should focus on the general process of information processing, especially the process of attention. In the current era characterized by an abundance of false information and emotionally charged “post-truth” narratives, it is imperative to focus on the universal processes of information processing, particularly the attentional processes, rather than confining the perspective solely to vaccine-specific information. This approach will enable the development of strategies to address this general process of information selection in the future. In other words, there should be a greater focus on investigating how individuals’ general processing of attentional information is influenced by various persuasive channels and media under the pressures of the spreading COVID-19 pandemic. The essence of persuasion, in this regard, hinges on captivating individuals’ attention toward information and steering their information selection accordingly. This necessitates that irrespective of the persuasion route employed, the audience should initially accord attention to the information disseminated by the source, thereby recognizing the attention effect of persuasive information. Although preceding studies have delved into the criteria influencing the follow-through and processing of information on social media, such as credibility, attitude consistency, and content richness (Vraga et al., 2016; Suelflow et al., 2019), there remains a paucity of research on the impact of persuasion pathways on the attention process (Kim et al., 2021).

Given that attention entails a rapid processing mechanism, traditional scales and self-reports are inadequate in capturing the nuanced process of information selection. Hence, employing event-related potentials (ERPs) with high temporal precision emerges as a judicious approach to examining such attention effects. This technique facilitates the exploration of the information processing timeline by analyzing real-time changes in EEG potentials, rendering it particularly apt for investigating the influence of persuasive information on attention processes.

### The LC4MP model

According to the Limited Capacity Model of Motivated Mediated Message Processing (LC4MP) posited by Lang (2000) and further elaborated by Lang and Bailey (2015), the capability for individual information processing is inherently bounded, with only a finite cache of cognitive resources at disposal for perception, encoding, comprehension, and memory tasks. In contrast, media presents a continuously variable and redundant stream of information, disseminated through a myriad of sensory channels (e.g., visual, auditory, tactile) and a diverse range of formats (e.g., verbal, textual, still imagery, moving imagery) (Lang, 2006). This inherent dichotomy necessitates that individuals allocate their constrained processing capacity to a select portion of the prevailing information, thereby rendering the efficacious functionality of attention-driven information selection mechanisms as imperative.

This model is an explanation of the basic process of information processing, and should also play a fundamental role in the process of persuasion. In the context of this model, then, it is important to consider the role of attention in the Elaboration Likelihood Model (ELM). According to this model, attention should play different roles in different persuasion routes: the central route requires careful thinking and understanding of information, in which information processors tend to invest more cognitive resources; on the other hand, the peripheral route has lower information processing requirements and only needs to process some surface and edge information without spending too much cognitive resources. That is, the two different persuasion paths should have different effects on the allocation of attention resources.

Therefore, it is necessary for us to adopt a way to investigate the role of this information selection process in the ELM model, and a good experimental paradigm in this regard is the visual search paradigm. This paradigm requires finding a target stimulus in different distractions, and contains two different search ways: parallel search that requires less cognitive resources and sequential search that requires more cognitive resources, making it an excellent choice to probe the cognitive resource allocation behind the two persuasion routes.

### Visual search task: unraveling attention and persuasion dynamics

In exploring the interaction of persuasion strategies within the Elaboration Likelihood Model, this study integrates the use of event-related potentials (ERPs) and a visual search task to capture the rapid and nuanced processes of attention and information selection. While traditional self-report measures often fall short in detecting intricate cognitive dynamics, the employment of ERPs proves invaluable, especially for examining the influence of central and peripheral routes in early attentional processing under diverse media conditions (Kim et al., 2021). Complementing this, the visual search task methodology, requiring participants to identify specific target stimuli amidst distractors, further refines our understanding of attentional processes (Hopfinger et al., 2004; Zhang and Wang, 2012; Yeh et al., 2019). This dual approach synergistically allows for a more precise exploration of how persuasive strategies impact cognitive engagement and attentional allocation, key aspects in the field of attention research.

In the trial where the target is present, the target is accompanied by a different number of distractors, while in the trial where the target is not present, only distractors are present. Participants are usually asked to do simple target detection, and the keystroke response indicates the presence or absence of the target, respectively, and the reaction time is usually the dependent variable of the measurement. In this study, we will initially present participants with central and peripheral route persuasion materials across different media (video or text), followed by instructing them to complete a visual search task. Reading tasks featuring different persuasive routes resemble a “priming” task, whereas the visual search task is akin to a “probing” task. To guard against response biases sensitive to vaccine information in the context of a pandemic, we employ a classic visual search task using neutral stimuli unrelated to vaccine information.

Predicated on the distinct stages of attention processing, two principal modalities of information processing emerge, namely parallel search and serial search. These correspond to automatic processing during the pre-attention phase and controlled processing during the attention phase, respectively (Treisman and Gelade, 1980; Snyder et al., 2012). Parallel search conforms to the processing of the pre-attention stage, that is, the target item can be found without attention. Serial search, on the other hand, requires attention to scan the location of each stimulus one by one, in order to determine whether the target item is present, and therefore the reaction time will be extended. In other words, whether attention is involved or not and how it is processed can be used to explain the differences between the two search conditions, and the differences between the two can be used to support the differences in the processing of attention at different stages. As for possible changes in ERPs components, previous studies have shown that early components in visual search, such as P1 and N1, represent selective attention (Munte et al., 1996) and are usually associated with enhanced attention in perceptual information processing. Moreover, the N1 amplitude reflects the recognition process within the focus of attention (Doallo et al., 2005). The P2, N2 and P3 components imply the adjustment of attention span, cognitive conflict, and allocation of attention resources (Gao et al., 2004; Sawaki and Katayama, 2008; Wang et al., 2016). In this study, because we are the first to explore the effects of different persuasion paths on attention, these components of visual search are likely to change, and the specific mechanisms are still unclear and need to be investigated.

The synthesis of this experimental paradigm with the event-related potentials (ERPs) technique paves the way for an implicit and real-time exploration of the temporal dynamics inherent to information selection processes. Consequently, this study is poised to scrutinize the ramifications of two disparate persuasion pathways on individuals’ attention efficacy. We expect that if the peripheral route consumes relatively little cognitive resources during the “priming” phase, then relatively more cognitive resources will be retained during the “probing” phase, and the opposite is the case for the central route. This difference in cognitive resource capacity would be reflected in the visual search task. It is postulated that the central and peripheral routes may exert differential modulatory effects on the ERP components within the ambit of the visual search task. That is, our priori hypothesis is that different persuasion routes and search types may have different effects on late components as indicators of cognitive resource allocation.

## Method

### Participant

The study utilized a three-factor mixed design, characterized by a 2 (media type: video vs. text) × 2 (persuasion route: central vs. peripheral) × 2 (search type: serial search vs. parallel search) matrix. Herein, media type and persuasion route functioned as between-participant factors, while search type operated as a within-participant factor.

Based on an *a priori* power analysis with *β* = 0.2, an effect size f of 0.4, and *α* = 0.05, it was estimated that that at least 73 participants are required for this study. Consequently, a total of 87 student participants at a northern China university, comprising 42 males and 45 females, were enlisted through WeChat and university forum channels, with ages ranging from 18 to 33 (*M* = 22.79, SD = 2.53) (see Table 1). Participants were randomly divided into four groups, delineated as Group VC: video & central group (11 males and 12 females), Group VP: video & peripheral group (11 males and 13 females), Group TC: text & central group (10 males and 10 females), and Group TP: text & peripheral group (10 males and 10 females). All participants were right-handed, possessing normal or corrected-to-normal vision, and exhibited no history of neurological afflictions or dependencies on tobacco or alcohol, nor were they currently under the influence of psychotropic medications. Prior to partaking in the experiment, all participants meticulously perused and endorsed the informed consent document, and were subsequently remunerated financially upon the culmination of the experiment.

**Table 1:** Demographic data of participants.

		Age				
Group	*N*	*M*	*SD*	*F*	*p*-value	*η^2^*
video & central (VC)	23	22.52	3.32	0.606	0.613	3.959
video & peripheral (VP)	24	22.54	1.87			
text & central (TC)	20	23.45	2.21			
text & peripheral (TP)	20	22.75	2.59			

The experiment procedures were approved by the sponsoring university’s institutional review board (Institutional Review Board of the State Key Laboratory of Cognitive Neuroscience and Learning, Beijing Normal University), each participant was paid about $15 and all the procedures for this study were performed in compliance with the principles of the Declaration of Helsinki.

### Materials

In our investigation into the effects of persuasion strategies on attention and information processing, we utilized a mixed-methods approach, incorporating both video and text formats to deliver content designed to engage participants through either central or peripheral routes of persuasion. This approach allows us to explore how different modalities and persuasion strategies influence cognitive engagement and attentional processes.

### Media format: video vs. text

#### Video materials

The video segments were crafted to provide a dynamic and multimodal experience, combining both visual and auditory elements. This modality leverages the human propensity for visual learning and the impact of auditory cues on emotional resonance. The video content was designed to simulate a news report, featuring a female anchor presenting information about COVID-19 vaccination. The inclusion of professional tone, body language, and background imagery was intended to mirror real-life news consumption experiences, thereby testing the efficacy of persuasive communication in a familiar format.

#### Text materials

The text-based materials, on the other hand, required participants to engage with written content, focusing solely on visual processing of the message. The texts were carefully structured to parallel the informational content of the videos, providing a direct comparison between the modalities. The written format allowed for an examination of how the absence of auditory and visual dynamics influences the processing of persuasive messages regarding COVID-19 vaccination.

### Persuasion strategy: central vs. peripheral persuasion

#### Central persuasion

Central persuasion materials were developed with an emphasis on logical argumentation and evidence-based appeals. The content was designed to encourage deep, reflective processing of the message’s merit, focusing on the efficacy and safety of COVID-19 vaccines. This route targets the audience’s capacity for analytical thinking, requiring active engagement with the content to form or change opinions based on the strength of the arguments presented.

#### Peripheral persuasion

In contrast, peripheral persuasion materials utilized cues that do not directly relate to the argument’s logical structure. Instead, these materials were aimed at influencing attitudes through emotional appeals, the credibility or attractiveness of the message source, and other context-specific cues. The peripheral route is predicated on the assumption that not all message processing involves active cognitive engagement with the content’s core arguments, relying instead on indirect factors to sway opinions.

#### Text central (TC) group

Participants in the Text Central group were presented with written materials designed to persuade through the central route. These materials were structured around logical arguments and evidence-based information regarding the importance and efficacy of COVID-19 vaccinations. The text for Group TC, titled“COVID-19 vaccination is a significant measure for the society pandemic control” encompassing 1,379 Chinese characters. This text, requiring approximately 3 to 5 min to read.

#### Text peripheral (TP) group

The Text Peripheral group received written materials that aimed to persuade through the peripheral route. This approach utilized emotional appeals rather than focusing solely on the argument’s logical structure. The text for Group TP, titled “This article tells you whether you should get COVID-19 vaccination or not” contained 1,435 Chinese characters, also necessitating a reading span of 3 to 5 min.

#### Video central (VC) group

Participants in the Video Central group were exposed to video content that employed central persuasion techniques. Similar to the TC group, the video was designed to present logical arguments and evidence supporting the effectiveness and safety of COVID-19 vaccinations. The video content had a duration of approximately 5 min and 16 s.

#### Video peripheral (VP) group

The Video Peripheral group viewed video materials tailored to persuade through peripheral cues. This group’s content mirrored the TP group’s strategy but within a video format, emphasizing emotional appeals. The video content had a duration of approximately 5 min and 49 s.

A preliminary Material Evaluation was conducted, scaling from 1 for central persuasion to 7 for peripheral persuasion, to ascertain the perceived persuasive nature of the materials. The results affirmed that materials for Groups VC and TC (*M* = 2.67, *SD* = 1.44) were predominantly perceived as central persuasion, while those for Groups VP and TP (*M* = 5.25, *SD* = 0.87) were inclined towards peripheral persuasion (*t* (11) = 4.33, *p* = 0.001), ensuring the distinct persuasive delineation of the materials.

### Manipulation check

After viewing the materials, participants from each group were asked to complete a set of Questions and Answers (Q&A) to ensure they grasped the main ideas of the content. This questionnaire consisted of six items related to the COVID-19 vaccination information presented in the materials, with a total possible score of 120. The average scores were as follows: Text Central (TC) Group achieved an average score of 92.10 (SD = 22.71), Text Peripheral (TP) Group scored 100.50 on average (SD = 20.36), Video Central (VC) Group had an average score of 82.48 (SD = 23.95), and Video Peripheral (VP) Group scored an average of 91.33 (SD = 21.65).

### Procedure

Participants participated in the experiment individually in quite room. Initially, a 3-min relaxation period was provided to ensure the stabilization of participants’ emotional states at baseline levels. Subsequently, participants from the four distinct groups engaged with specified news materials either through viewing or reading. Thereafter, they were prompted to respond to pertinent questions aimed at fostering a deeper engagement with the materials (e.g., *Could you please clarify the overarching theme of this article? How many vaccines are mentioned in the article? Could you list the names of the* var*ious vaccines that are mentioned?*). Following this, participants were guided to complete the visual search task. Finally, a subsequent 3-min relaxation period was afforded to assist in restoring participants’ emotional states to baseline levels. The cumulative duration of the task sequence approximated 25 min.

**Figure 1:**
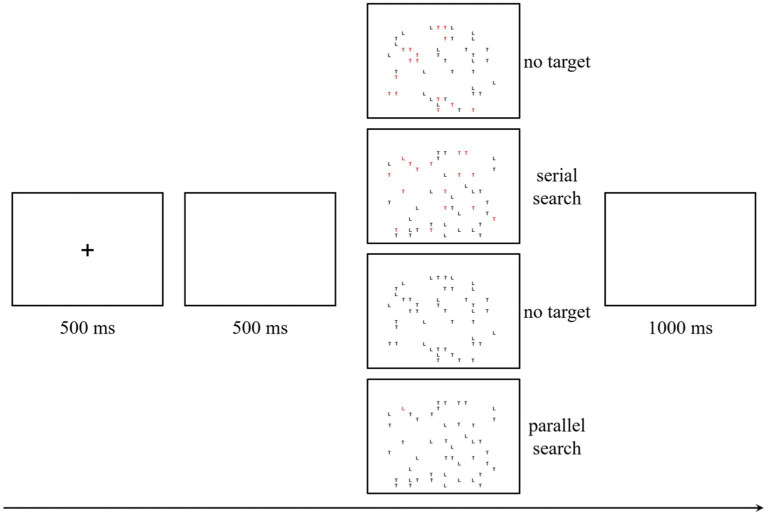
The procedure of the visual search task.

In the visual search task, stimuli were randomized and presented using E-Prime 3.0 software. Each trial initiated with a 500 ms grey fixation appearing at the screen’s center, serving as a cue for participants to prepare and maintain their gaze at the screen’s midpoint. Following a 500 ms interval of a blank screen, the stimulus image was displayed. This image comprised an array of the letters “T” and “L,” with the red “L” serving as the target stimulus and the “T” acting as the distractor. Four conditions were delineated: in the first condition, no target stimuli were present, and the array included 15 red “T”s, 15 black “T”s, and 15 black “L”s; in the second condition, aligned with the serial search paradigm, a single target stimulus was included amidst 15 red “T”s, 15 black “T”s, and 15 black “L”s; in the third condition, devoid of target stimulus or red distractor, the arrangement consisted of 30 black “T”s and 15 black “L”s; in the fourth condition, adhering to the parallel search paradigm, a singular target stimulus was accompanied by 30 black “T”s and 15 black “L”s. Participants were tasked with determining the presence or absence of a red “L” within the image. In cases where a red “L” was detected, participants were instructed to press the “F” key on the keyboard, whereas absence of the red “L” warranted pressing the “J” key. The stimulus interface was presented until the participant pressed a key and then disappeared. Upon key press, a blank screen ensued for a 1,000 ms duration, after which the program autonomously transitioned to the ensuing trial. The outlined procedure is depicted in Figure 1. Each condition encompassed 20 unique images, aggregating to a total of 80 images throughout the task.

Prior to the main experiment, a practice block comprising 20 trials was conducted to familiarize participants with the task. The formal experiment encompassed two blocks, each consisting of 80 trials, culminating in a total of 160 trials. Participants could rest for a while between the blocks. Concurrent with the behavioral task during the formal experiment, the event-related potential (ERP) data of the participants were captured.

The stimuli were rendered on a Lenovo 23-inch CRT computer monitor, boasting a refresh rate of 100 Hz and a resolution of 1920 × 1,080 pixels. The viewing distance, measured from the eye to the screen, was approximately 65 cm. Each image spanned a visual angle of 44.89° × 25.25°. We calculated the accuracy and the reaction time of the correct reaction.

### ERP data acquisition and processing

The EEG data was captured utilizing a Neuroscan SynAmps^2^ 64-channel recording system (Compumedics Ltd., Australia). During the AC online recording phase, the reference electrode was positioned at the left mastoid, and was later converted to the average reference of bilateral mastoids during offline analysis. Both the vertical electrooculogram (VEOG) and horizontal electrooculogram (HEOG) were documented via bipolar recording. The VEOG electrodes were situated above and below the midpoint of the left orbit, whereas the HEOG electrodes were positioned outboard of the left and right lateral canthus. The impedance for all electrodes was maintained below 10 kΩ. The filter bandpass ranged from 0.05 Hz to 200 Hz, with a sampling frequency set at 1000 Hz.

The pre-processing and analysis of the data were executed using EEGLAB 14.1.1, a toolbox within MATLAB2016a. EEG data were subjected to a bandpass filter within a range of 0.5–30 Hz. Artifacts encompassing blinks and EMG were rectified offline through Independent Component Analysis (ICA). Epochs were delineated for a specified duration (−100 ms to 500 ms relative to stimulus onset), and a baseline correction was administered within the 100 ms pre-stimulus window. Any epochs with amplitude values surpassing ±100 *μ*V were manually discarded as artifacts, albeit the number of valid trials in each experimental condition exceeded 30. Mean values were computed for both serial and parallel search conditions. Based on Milne et al. (2013), Niu et al. (2008), and previous experience, electrodes of interest were identified as P3, P1, Pz, P2, P4, PO3, POz, PO4, O1, Oz, and O2, and their respective averages were designated as the dependent variables in the statistical evaluation. Upon meticulous examination of the entire waveforms, the temporal windows of five components were delineated as follows: P1: 70–100 ms, N1: 100–180 ms, P2: 180–240 ms, N2: 240–310 ms, P3: 310–430 ms. The statistical analysis was facilitated by IBM SPSS 22.0. The Greenhouse–Geisser correction was employed on the *p*-values, and subsequent *post hoc* examinations were carried out utilizing pairwise comparisons with a Bonferroni correction. The reason for using Greenhouse–Geisser is because ERPs belong to near field potentials which are more concentrated locally than elsewhere, thus not satisfying spherical hypothesis well and requiring correction. This information can be found in Luck (2014).

## Results

### Reaction times (RTs) of visual search

**Figure 2:**
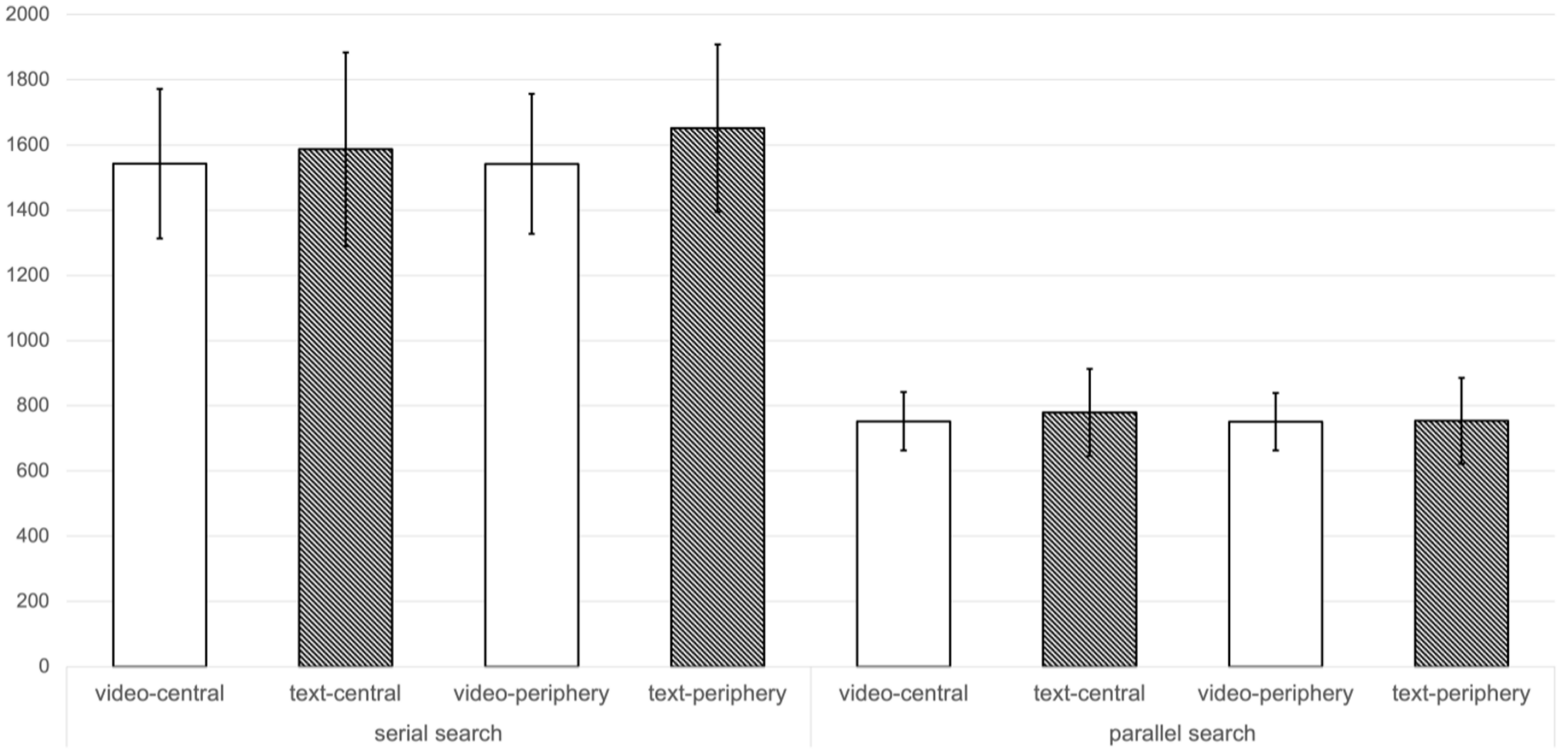
Means (and Standard Errors) in average reaction time of the visual search tasks across the four groups (*N* = 87).

Figure 2 delineates the reaction times associated with the visual search task across the four groups. A three-way Analysis of Variance (ANOVA) was conducted on the reaction time data, revealing a significant main effect solely attributed to the search type (*F* (1, 83) = 1400.834, *p* < 0.001, partial *η*^2^ = 0.944), the RTs of parallel search is faster than that of serial search. In contrast, the main effect of the persuasion method proved to be insignificant (*F* (1, 83) = 0.070, *p* = 0.791, partial *η^2^* = 0.001), as was the main effect of media type (*F* (1, 83) = 1.714, *p* = 0.194, partial *η^2^* = 0.020). The interactions among these factors were also found to be non-significant. For a detailed breakdown of the interactions, see Table 2.

**Table 2:** Accuracy (ACC; %) and reaction time (RT; ms) of visual search task in the four groups (*N* = 87).

		Serial search		Parallel search	
		Video	Text	Video	Text
Central	RT	1542.42 ± 229.13	1586.88 ± 296.94	752.03 ± 89.39	779.13 ± 133.49
	ACC	0.89 ± 0.09	0.89 ± 0.07	0.93 ± 0.05	0.94 ± 0.03
Periphery	RT	1541.88 ± 214.62	1651.60 ± 256.49	750.75 ± 87.73	753.53 ± 131.74
	ACC	0.91 ± 0.08	0.90 ± 0.08	0.91 ± 0.06	0.90 ± 0.077

### Accuracy (ACC) of visual search

A three-way Analysis of Variance (ANOVA) was conducted on the accuracy data, revealing a significant main effect solely attributed to the search type (*F* (1, 83) = 7.687, *p* = 0.007, partial *η^2^* = 0.085), and the ACC of serial search is higher than that the ACC of parallel search. Conversely, the main effect of the media type proved to be insignificant (*F* (1, 83) = 0.037, *p* = 0.848, partial *η^2^* = 0.000), as was the main effect of persuasion way (*F* (1, 83) = 0.352, *p* = 0.555, partial *η^2^* = 0.004). The interaction effect of the two groups was insignificant (*F* (1, 83) = 0.172, *p* = 0.680, partial *η^2^* = 0.002). The interaction of search type × persuasion way is significant (*F* (1, 83) = 5.212, *p* = 0.025, partial *η2* = 0.059). Through further simple effect analysis of search type × persuasion way, it is found that the persuasion way of central is significantly larger than that in the persuasion way of periphery in the parallel search task (*p* = 0.029). However, in the serial search task, there is no significant difference between persuasion way of central and persuasion way of periphery (*p* = 0.410). The interactions among all the remaining factors were found to be non-significant. For a detailed breakdown of the interactions, see Supplementary Tables S3, S4.

### ERPs results

**Figure 3:**
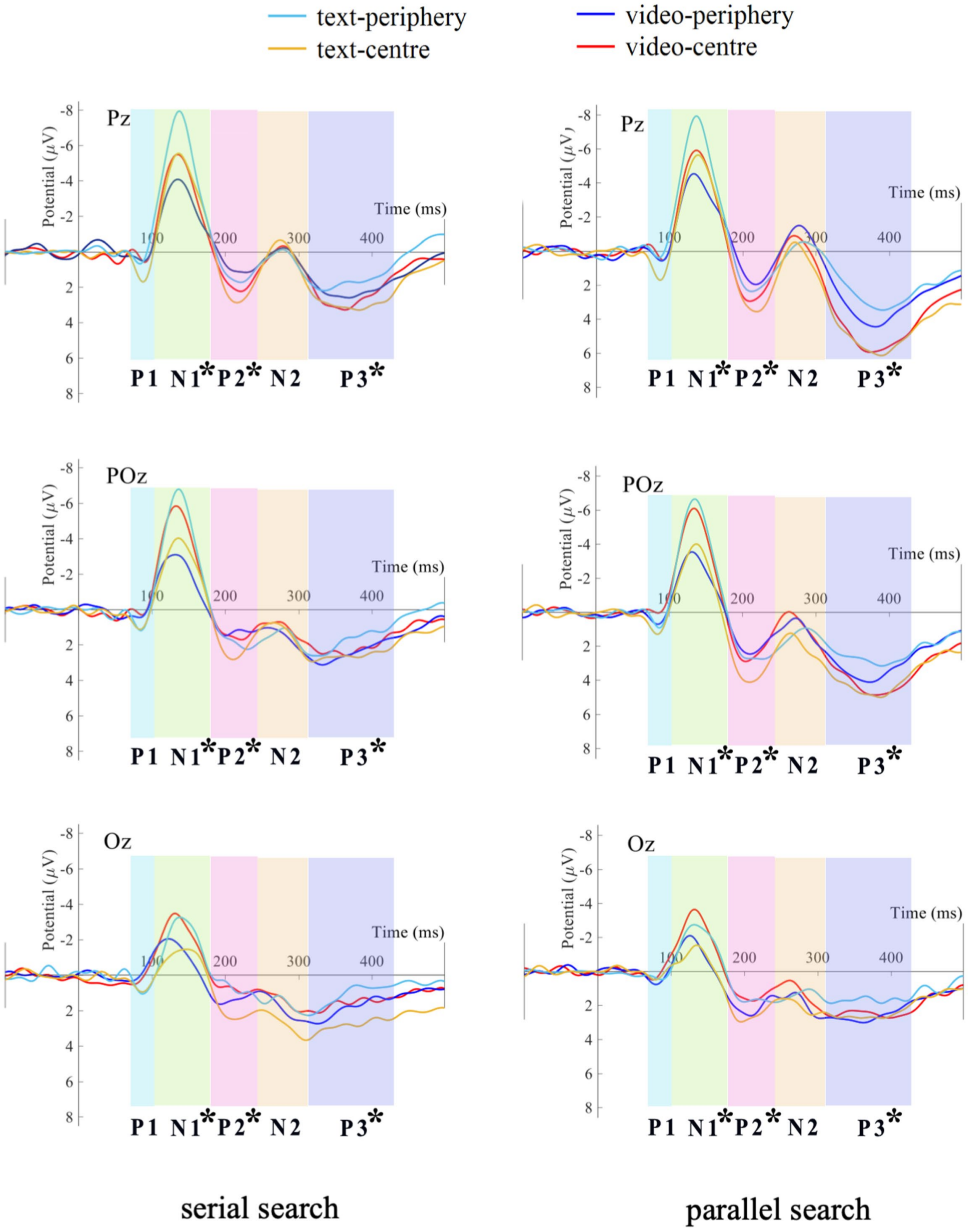
Grand averaged ERPs of serial search and parallel search.

Note: On the N1 component, the interaction of persuasion way × media type is significant. On the P2 component, the main effect of search type is significant. On the P3 component, the main effect of search type is significant.

The grand averaged waveforms at Pz, POz, and Oz of serial search and parallel search are shown in Figure 3. The corresponding ERP scalp topographies are included in Supplementary Figures S4–S8. Additionally, the grand averaged ERP waveforms across these electrodes (Pz, POz, Oz) are depicted in Supplementary Figure S9.

Topographic maps of the five components of the visual search task: P1, N1, P2, N2 and P3 are shown in Supplementary Figures S4–S8, and a summary of all statistical results can be found in Supplementary Table S3.

Three-way repeated ANOVA was adopted to, respectively, analyze the amplitude of P1, N1, P2, N2, and P3 of the visual search task, in a 2 (search type: serial search vs. parallel search) × 2 (persuasion way: central vs. periphery) × 2 (media type: video vs. text) mixed design. Media type (video vs. text) and persuasion way (central vs. periphery) are between-subject factors, and search type (serial search vs. parallel search) is within-subject factor. The results showed that (detailed results are shown in Supplementary Table S5).

On the P1 component, the main effect of search type is not significant (*F* (1, 83) = 0.034, *p* = 0.854, partial *η^2^* < 0.001). The main effect of persuasion way is not significant (*F* (1, 83) = 0.117, *p* = 0.773, partial *η^2^* = 0.001). The main effect of media type is not significant (*F* (1, 83) = 1.154, *p* = 0.286, partial *η^2^* = 0.014). The interaction of search type × persuasion way is not significant (*F* (1, 83) = 0.187, *p* = 0.667, partial *η^2^* = 0.002). The interaction of search type × media type is not significant (*F* (1, 83) = 0.203, *p* = 0.653, partial *η^2^* = 0.002). The interaction of persuasion way × media type is not significant (*F* (1, 83) = 0.634, *p* = 0.428, partial *η^2^* = 0.008). The interaction of search type × persuasion way × media type is not significant (*F* (1, 83) = 1.830, *p* = 0.180, partial *η^2^* = 0.022).

On the N1 component, the main effect of search type is not significant (*F* (1, 83) = 0.011, *p* = 0.916, partial *η^2^* < 0.001). The main effect of persuasion way is not significant (*F* (1, 83) = 0.337, *p* = 0.563, partial *η^2^* = 0.004). The main effect of media type is not significant (*F* (1, 83) = 0.310, *p* = 0.579, partial *η^2^* = 0.004). The interaction of search type × persuasion way is not significant (*F* (1, 83) = 0.366, *p* = 0.547, partial *η^2^* = 0.004). The interaction of search type × media type is not significant (*F* (1, 83) = 1.296, *p* = 0.258, partial *η^2^* = 0.015). The interaction of persuasion way × media type is significant (*F* (1, 83) = 5.805, *p* = 0.018, partial *η^2^* = 0.065). Then, through further simple effect analysis of persuasion way × media type, it is found that the amplitude of N1 component in the persuasion way of periphery is significantly larger than that in the persuasion way of central in text media (*p* = 0.045). However, in video media, there is no significant difference between persuasion way of central and persuasion way of periphery (*p* = 0.181). The interaction of search type × persuasion way × media type is not significant (*F* (1, 83) = 1.067, *p* = 0.796, partial *η^2^* = 0.001).

On the P2 component, the main effect of search type is significant (*F* (1, 83) = 31.781, *p* < 0.001, partial *η^2^* = 0.277), and the amplitude of P2 component in the parallel search is larger than that in the serial search. The main effect of persuasion way is not significant (*F* (1, 83) = 1.057, *p* = 0.307, partial *η^2^* = 0.013). The main effect of media type is not significant (*F* (1, 83) = 1.060, *p* = 0.306, partial *η^2^* = 0.013). The interaction of search type × persuasion way is not significant (*F* (1, 83) = 0.967, *p* = 0.328, partial *η^2^* = 0.012). The interaction of search type × media type is not significant (*F* (1, 83) = 0.418, *p* = 0.520, partial *η^2^* = 0.005). The interaction of persuasion way × media type is not significant (*F* (1, 83) = 0.648, *p* = 0.423, partial *η^2^* = 0.008). The interaction of search type × persuasion way × media type is not significant (*F* (1, 83) = 0.115, *p* = 0.736, partial *η^2^* = 0.001).

On the N2 component, the main effect of search type is not significant (*F* (1, 83) = 0.954, *p* = 0.332, partial *η^2^* = 0.011). The main effect of the persuasion way is not significant (*F* (1, 83) = 0.031, *p* = 0.861, partial *η^2^* = 0.013). The main effect of the media type is not significant (*F* (1, 83) = 0.505, *p* = 0.479, partial *η^2^* = 0.005). The interaction of search type × persuasion way is not significant (*F* (1, 83) = 2.247, *p* = 0.138, partial *η^2^* = 0.026). The interaction of search type × media type is not significant (*F* (1, 83) = 1.968, *p* = 0.5164, partial *η^2^* = 0.023). The interaction of persuasion way × media type is not significant (*F* (1, 83) = 0.411, *p* = 0.523, partial *η^2^* = 0.005). The interaction of search type × persuasion way × media type is not significant (*F* (1, 83) = 0.198, *p* = 0.657, partial *η^2^* = 0.002).

On the P3 component, the main effect of search type is significant (*F* (1, 83) = 36.907, *p* < 0.001, partial *η^2^* = 0.308), and the amplitude of P3 component in the parallel search is larger than that in the serial search. The main effect of persuasion way is significant (*F* (1, 83) = 4.142, *p* = 0.045, partial *η^2^* = 0.048), and the amplitude of P3 component in the persuasion way of central is larger than that in the persuasion way of periphery. The main effect of media type is not significant (*F* (1, 83) = 0.038, *p* = 0.847, partial *η^2^* < 0.001). The interaction of search type × persuasion way is significant (*F* (1, 83) = 5.650, *p* = 0.020, partial *η^2^* = 0.064). Through further simple effect analysis of search type × persuasion way, it is found that the amplitude of P3 component in the persuasion way of central is significantly larger than that in the persuasion way of periphery in the parallel search task (*p* = 0.013). However, in the serial search task, there is no significant difference between persuasion way of central and persuasion way of periphery (*p* = 0.212). The interaction of search type × media type is not significant (*F* (1, 83) = 0.416, *p* = 0.521, partial *η^2^* = 0.005). The interaction of persuasion way × media type is not significant (*F* (1, 83) = 1.112, *p* = 0.295, partial *η^2^* = 0.013). The interaction of search type × persuasion way × media type is not significant (*F* (1, 83) = 0.019, *p* = 0.890, partial *η^2^* < 0.001).

## Discussion

This study attempts to examine the effects of two different persuasion ways in the ELM model on the information processing mechanisms of different attentional searches. The results demonstrated that employing the peripheral persuasion method through text media engendered a more pronounced wave of N1 components in comparison to the central persuasion method, indicating a response to earlier phases of attentional processing. On the contrary, the influence exerted by both persuasion methods on N1 components remained consistent when video media was utilized. In subsequent stages, as represented by P2 and P3 components, the amplitude of parallel search notably exceeded that of serial search. Moreover, there was no difference in P3 component between the two persuasion methods in the serial search task; on parallel search tasks, P3 on the central method is larger than P3 on the peripheral method. In terms of behavioral response, the data on response time repeat the classical result that parallel search is faster than serial search. In terms of accuracy, in addition to the higher accuracy of sequential search, it is also found that the central persuasion route in parallel search tasks has a higher accuracy.

First of all, there are relatively consistent findings on the reaction time and accuracy results of the search type: parallel search has shorter reaction time and higher accuracy, which is due to the nature of the search task itself. Parallel search is a kind of “pop-up” phenomenon, and individuals can easily find the target without investing a lot of cognitive resources. A more interesting result is the interaction between the search types and the persuasion ways. We find no difference between the two strategies in serial search; however, in parallel search, the search accuracy influenced by the central persuasion path is significantly higher than that influenced by the peripheral path. This may indicate the different effects of the two persuasion ways on the automatic allocation of attention resources. Sometimes, behavioral data may not reveal differences, making it necessary to further discuss ERP results.

Previous studies have illustrated that N1 is often associated with enhanced attention towards perceptual information processing, and that N1 amplitude mirrors recognition processes within the focal point of attention (Doallo et al., 2005). Alternatively, N1 primarily reflects the augmentation of sensory processing during early attention stages and the processing of discriminative distinctions in visual information processing. It is widely accepted that wave amplitude is proportional to the number of neuronal activations, epitomizing the intensity of mental load during information processing (Hillyard and Anllo-Vento, 1998). Higher wave amplitude signifies more extensive brain area involvement in sensory information processing, and the wave amplitude escalates with the increase in attentional energy allocation (Luck, 1995).

In summary, this process illustrates the early selection of information by attentional mechanisms. In the present study, the peripheral persuasion method was found to enhance the early attentional process of text media. Compared to video media, text media is a medium that necessitates more profound processing (Lang, 2000, 2006; Lang and Bailey, 2015), and hence requires more attention resources under the constraint of limited resources, which leads to larger N1 amplitudes.

Consequently, we posit that one distinction between peripheral and central methods at the microscopic and rapid cognitive neural level lies in their differing attentional mechanisms, with peripheral method eliciting early attentional selection processes stronger, and central method initiating them relatively weaker. This finding aligns with the biased competition model of visual attention proposed by Desimone and Duncan (1995), wherein top-down attentional control, influenced chiefly by subjective factors such as goals and expectations, plays a pivotal role in the attentional selection process. Hence, we can further speculate that these subjective factors, which lead to cognitive competition, may underpin the mechanism behind the influence of persuasion methods on information selection—a prospect that warrants deeper exploration in future studies.

Another interesting finding of this study emerged from the P2 and P3 components associated with late attentional processing. During these processing stages, no distinctions were observed between the media type factors. It is generally held that the P2 component reflects the adjustment of attentional span within visual space (Gao et al., 2004; Wang et al., 2016), while the P3 component serves as an electrophysiological marker of attentional allocation (Sawaki and Katayama, 2008). The observation that the amplitudes of the P2 and P3 components in the parallel search were more pronounced compared to the serial search is unsurprising. What is interesting, however, is the interaction between persuasion ways and search types. Simple effect analysis shows that there is no difference between the two persuasion ways in the serial search task. On parallel search tasks, P3 on the central method is larger than P3 on the peripheral method. This is consistent with the previous results for the accuracy rate. This means that in the later stages of attention, the central method begins to affect the allocation of attention resources. This further confirms the previous results of N1, that the peripheral path causes more of the early attention selection process, while the central path influences more of the late attention selection process. In a way, the two findings are complementary. In addition, the occurrence time of these effects did not exceed 500 ms, that is to say, the audience’s selection of persuasion method represents an “instantaneous effect.” Individuals tend to form a quick impression of persuasive information within a brief timeframe and base subsequent judgments and decisions on this impression, which might be an adaptive mechanism shaped by long-term evolution.

### Possible drawbacks and limitations

Here, considering other possible explanations for these findings, we also need to draw the reader’s attention to some possible limitations to address and remedy critical points.

The first is about the between-subject experimental design adopted in this study. An inherent weakness of this design is the inability to control for individual differences, since the two groups of participants may already be different, and the results of the comparison between the two groups will be confused with these differences. However, this does not justify dismissing the experimental design altogether. On the contrary, using a within-subject design in this study would have resulted in more serious consequences due to potential practice and fatigue effects from repeatedly presenting highly consistent stimuli in different media. Additionally, considering different persuasion routes within subjects could lead to disastrous results (as participants would view the material four times and complete a visual search task each time). Therefore, we can only consider the search type as a within-subject factor in the ERPs task, while treating the other two independent variables as between-subject factors.

Another, more important aspect is that our interpretation of the N1 results focuses primarily on the allocation of attention resources. As mentioned earlier, we believe that peripheral route triggers the early attention selection process more strongly, while central route initiates them with relatively less intensity (earlier N1 is larger under the peripheral route and later P3 is larger under the central route, ignoring everything else). According to the attentional resource theory, the larger N1 in the probe phase can be explained by the relatively small number of attentional resources consumed during peripheral reading before the search task, while the larger amount of attention consumed during central reading (less attention to search after central reading, i.e., a spillover from prime to the search task).

But on the other hand, since the peripheral persuasion approach mainly acts on subjective factors such as audience perception, emotion and motivation, these factors further compete for cognitive resources and compete with the expected cognitive resources required for text-mediated processing. The N1 component is more sensitive to emotion-related information (Scott et al., 2009; Wang et al., 2015; Leveille et al., 2022), will also lead to an increase in N1 amplitude. This may make some readers confused: is the increase in N1 amplitude caused by text media in the peripheral route due to the retention of more attentional spillover resources (attentional spillover)? Or is it an emotional spillover caused by emotions “spilling” from the prime phase (emotional spillover)? We think the former is more likely, and the key lies in the form of media. The difference we found was mainly in the text media (the N1 amplitude of the light blue line representing “text-peripheral” was larger than the N1 amplitude of the yellow line representing “text-central,” see in Figure 3). As mentioned earlier, compared to video media, text media is a medium that necessitates more profound processing. The allocation of attention resources plays a leading role. If it is an emotional spillover effect, then a similar effect should be observed in video media (the peripheral amplitude is larger than the central one). On the contrary, this pattern did not appear in the video media. In fact, a direct observation of the ERPs waveform shows the opposite trend (the N1 amplitude of the dark blue line representing the “video-peripheral” is slightly smaller than that of the red line representing the “video-central,” see in Figure 3), although it is not statistically significant. Therefore, while there may be some residual emotional spillover effect during the probe phase, it is not large and does not trump the attention spillover effect in cognitive competition. For the exploration of this question, further evidence is needed to clarify it in the future.

Finally, some readers may be interested in the relationship between the N1 effect and the P3 effect. We tend to think that with less cognitive engagement, the peripheral route consumes relatively fewer cognitive resources in the prime phase, so the more cognitive resources retained in the probe phase, the more N1 components can be induced in the early selection phase, and these resources are depleted by N1 in the later phase, so P3 can allocate fewer resources and its amplitude is smaller. While the central route consumes relatively more cognitive resources in the prime stage, there is no large N1 component in the early selection processing that first appears in the probe stage, and with the passage of time, attention resources gradually have a chance to recover, then resources can be allocated in the late stage, and P3 is produced.

At last, the current study identified immediate effects of differing persuasion methods on information selection mechanisms, potentially stemming from cognitive competition induced by various subjective factors under the constraint of limited attentional resources. However, this study only explored the timeline of this immediate effect without delving into its exact neural locus, due to the limitations posed by the research tools utilized. Future investigations may benefit from employing in-depth brain imaging techniques such as fMRI or NIRS. Additionally, the sample demographic in this study did not encompass all age groups, hence further research could expand upon different groups with diverse demographic characteristics such as gender and age. More crucially, effective persuasion towards vaccine acceptance should direct individuals’ attention towards accurate vaccine information, thereby having a facilitative effect on their information selection process, i.e., the attention process.

## Data availability statement

The original contributions presented in the study are included in the article/Supplementary material, further inquiries can be directed to the corresponding author.

## Ethics statement

The studies involving humans were approved by Institutional Review Board (IRB) at National Key Laboratory of Cognitive Neuroscience and Learning, Beijing Normal University. The studies were conducted in accordance with the local legislation and institutional requirements. The participants provided their written informed consent to participate in this study.

## Author contributions

LX: Conceptualization, Data curation, Funding acquisition, Methodology, Validation, Writing – original draft, Writing – review & editing. XC: Conceptualization, Data curation, Formal analysis, Investigation, Methodology, Project administration, Supervision, Validation, Writing – review & editing. LM: Formal analysis, Investigation, Methodology, Conceptualization, Writing – original draft. EZ: Data curation, Formal analysis, Visualization, Conceptualization, Methodology, Writing – original draft. GY: Conceptualization, Funding acquisition, Project administration, Supervision, Validation, Writing – review & editing.
