# Supplementary material for: Unveiling the influence of persuasion strategies on cognitive engagement: an ERPs study on attentional search
Source: Front Behav Neurosci. 2024 Sep 10;18:1302770. doi: 10.3389/fnbeh.2024.1302770 (PMC11420015; doi:10.3389/fnbeh.2024.1302770)
Supplement: Supplementary file 1 [file Data_Sheet_1.zip › Supplementary Materials/Table┬á5_Confirmed.docx]

**Table 5.**Three-way repeated ANOVA results of P1, N1, P2, N2 and P3

|  | *F* | *p* | partial *η*^2^ |
| --- | --- | --- | --- |
| P1 |  |  |  |
| search type | 0.034 | 0.854 | < 0.001 |
| persuasion way | 0.117 | 0.773 | 0.001 |
| media type | 1.154 | 0.286 | 0.014 |
| search type × media type | 0.203 | 0.653 | 0.002 |
| search type × persuasion way | 0.187 | 0.667 | 0.002 |
| media type × persuasion way | 0.634 | 0.428 | 0.008 |
| search type × media type × persuasion way | 1.830 | 0.180 | 0.022 |
| N1 |  |  |  |
| search type | 0.011 | 0.916 | < 0.001 |
| persuasion way | 0.337 | 0.563 | 0.004 |
| media type | 0.310 | 0.579 | 0.004 |
| search type × media type | 1.296 | 0.258 | 0.015 |
| search type × persuasion way | 0.366 | 0.547 | 0.004 |
| media type × persuasion way | 5.805 | 0.018 | 0.065 |
| search type × media type × persuasion way | 1.067 | 0.796 | 0.001 |
| P2 |  |  |  |
| search type | 31.781 | < 0.001 | 0.277 |
| persuasion way | 1.057 | 0.307 | 0.013 |
| media type | 1.060 | 0.306 | 0.013 |
| search type × media type | 0.418 | 0.520 | 0.005 |
| search type × persuasion way | 0.967 | 0.328 | 0.012 |
| media type × persuasion way | 0.648 | 0.423 | 0.008 |
| search type × media type × persuasion way | 0.115 | 0.736 | 0.001 |
| N2 |  |  |  |
| search type | 0.954 | 0.332 | 0.011 |
| persuasion way | 0.031 | 0.861 | 0.013 |
| media type | 0.505 | 0.479 | 0.005 |
| search type × media type | 1.968 | 0.164 | 0.023 |
| search type × persuasion way | 2.247 | 0.138 | 0.026 |
| media type × persuasion way | 0.411 | 0.523 | 0.005 |
| search type × media type × persuasion way | 0.198 | 0.657 | 0.002 |
| P3 |  |  |  |
| search type | 36.907 | < 0.001 | 0.308 |
| persuasion way | 4.142 | 0.045 | 0.048 |
| media type | 0.038 | 0.847 | < 0.001 |
| search type × media type | 0.416 | 0.521 | 0.005 |
| search type × persuasion way | 5.650 | 0.020 | 0.064 |
| media type × persuasion way | 1.112 | 0.295 | 0.013 |
| search type × media type × persuasion way | 0.019 | 0.890 | < 0.001 |
