# Supplementary material for: Unveiling the influence of persuasion strategies on cognitive engagement: an ERPs study on attentional search
Source: Front Behav Neurosci. 2024 Sep 10;18:1302770. doi: 10.3389/fnbeh.2024.1302770 (PMC11420015; doi:10.3389/fnbeh.2024.1302770)
Supplement: Supplementary file 1 [file Data_Sheet_1.zip › Supplementary Materials/Table┬á3_Confirmed.docx]

**Table 3.** Three-way ANOVA results of visual research task RTs

|  | *F* | *p* | partial *η*^2^ |
| --- | --- | --- | --- |
| search type | 1400.834 | < 0.001 | 0.944 |
| persuasion way | 0.070 | 0.791 | 0.001 |
| media type | 1.714 | 0.194 | 0.020 |
| search type × media type | 2.004 | 0.161 | 0.024 |
| search type × persuasion way | 1.075 | 0.303 | 0.013 |
| media type × persuasion way | 1.040 | 0.311 | 0.012 |
| search type × media type × persuasion way | 0.085 | 0.772 | 0.001 |
