# Supplementary material for: Unveiling the influence of persuasion strategies on cognitive engagement: an ERPs study on attentional search
Source: Front Behav Neurosci. 2024 Sep 10;18:1302770. doi: 10.3389/fnbeh.2024.1302770 (PMC11420015; doi:10.3389/fnbeh.2024.1302770)
Supplement: Supplementary file 1 [file Data_Sheet_1.zip › Supplementary Materials/Table 1_Confirmed.docx]

Table 1 Demographic data of participants

|  |  | Age | |  |  |  |
| --- | --- | --- | --- | --- | --- | --- |
| Group | n | M | SD | *F* | *p* | *η*^2^ |
| video & central (VC) | 23 | 22.52 | 3.32 | 0.606 | 0.613 | 3.959 |
| video & peripheral (VP) | 24 | 22.54 | 1.87 |  |  |  |
| text & central (TC) | 20 | 23.45 | 2.21 |  |  |  |
| text & peripheral (TP) | 20 | 22.75 | 2.59 |  |  |  |

|  |  | Group | | | |  |  |
| --- | --- | --- | --- | --- | --- | --- | --- |
|  | item | video & central (VC) | video & peripheral (VP) | text & central (TC) | text & peripheral (TP) | χ² | p |
| Gender | male | 11 | 11 | 10 | 10 | 0.107 | 0.991 |
|  | female | 12 | 13 | 10 | 10 |  |  |
| Personality | need for cognition | 13 | 12 | 10 | 11 | 0.305 | 0.959 |
|  | need for affect | 10 | 12 | 10 | 9 |  |  |
