# Supplementary material for: Unveiling the influence of persuasion strategies on cognitive engagement: an ERPs study on attentional search
Source: Front Behav Neurosci. 2024 Sep 10;18:1302770. doi: 10.3389/fnbeh.2024.1302770 (PMC11420015; doi:10.3389/fnbeh.2024.1302770)
Supplement: Supplementary file 1 [file Data_Sheet_1.zip › Supplementary Materials/Table 4_Confirmed.docx]

**Table 4.** Three-way ANOVA results of visual research task ACC

|  | *F* | *p* | partial *η*^2^ |
| --- | --- | --- | --- |
| search type | 7.687 | 0.007 | 0.085 |
| persuasion way | 0.352 | 0.555 | 0.004 |
| media type | 0.037 | 0.848 | 0.000 |
| search type × media type | 0.428 | 0.515 | 0.005 |
| search type × persuasion way | 5.212 | 0.025 | 0.059 |
| media type × persuasion way | 0.172 | 0.680 | 0.002 |
| search type × media type × persuasion way | 0.066 | 0.798 | 0.001 |
