# Supplementary material for: Unveiling the influence of persuasion strategies on cognitive engagement: an ERPs study on attentional search
Source: Front Behav Neurosci. 2024 Sep 10;18:1302770. doi: 10.3389/fnbeh.2024.1302770 (PMC11420015; doi:10.3389/fnbeh.2024.1302770)
Supplement: Supplementary file 1 [file Data_Sheet_1.zip › Supplementary Materials/Table 2_Confirmed.docx]

**Table 2.**

Accuracy(ACC;%) and reaction time(RT;ms) of visual search task in the four groups (N = 87)

|  |  | serial search | | parallel search | |
| --- | --- | --- | --- | --- | --- |
|  |  | video | text | video | text |
| central | RT | 1542.42 ± 229.13 | 1586.88 ± 296.94 | 752.03 ± 89.39 | 779.13 ± 133.49 |
|  | ACC | 0.89 ± 0.09 | 0.89 ± 0.07 | 0.93 ± 0.05 | 0.94 ± 0.03 |
| periphery | RT | 1541.88 ± 214.62 | 1651.60 ± 256.49 | 750.75 ± 87.73 | 753.53 ± 131.74 |
|  | ACC | 0.91 ± 0.08 | 0.90 ± 0.08 | 0.91 ± 0.06 | 0.90 ± 0.077 |
