# Supplementary figures and images for: Unveiling the influence of persuasion strategies on cognitive engagement: an ERPs study on attentional search
Source: Front Behav Neurosci. 2024 Sep 10;18:1302770. doi: 10.3389/fnbeh.2024.1302770 (PMC11420015; doi:10.3389/fnbeh.2024.1302770)

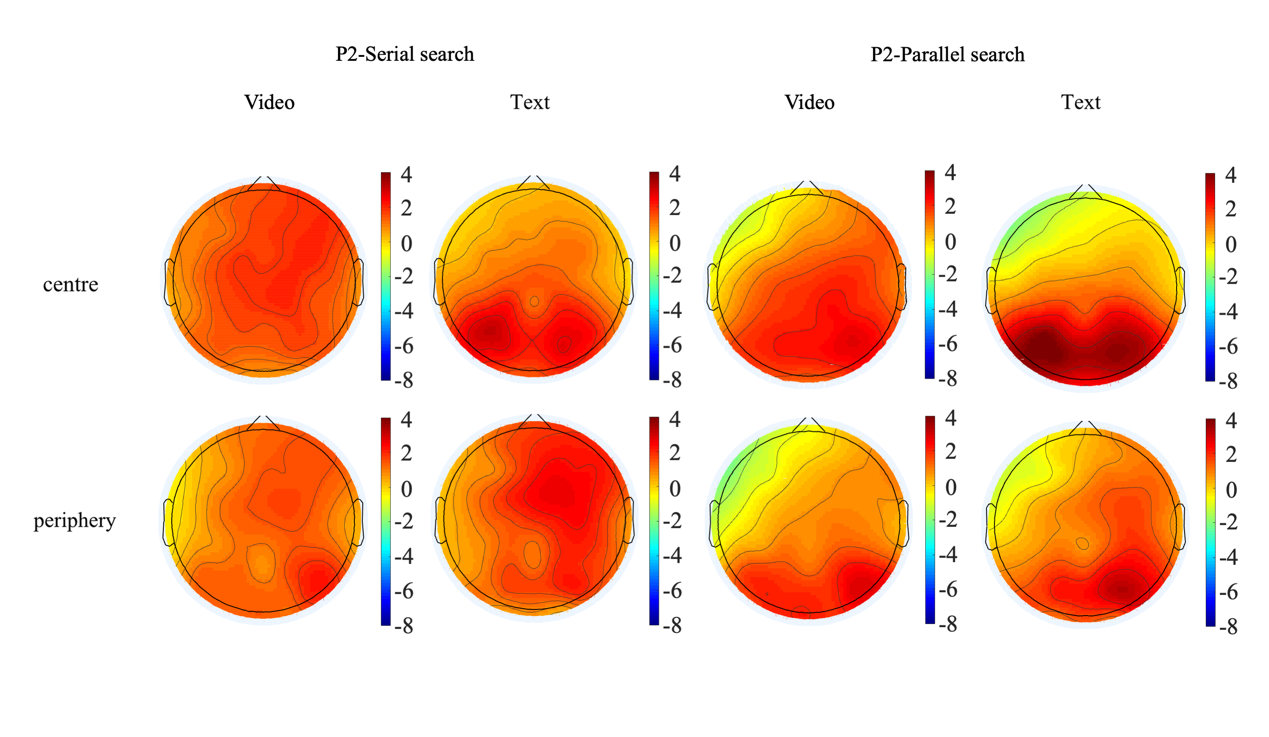

Supplement: Supplementary file 1 [file Data_Sheet_1.zip › Supplementary Materials/Figure 6.tif]

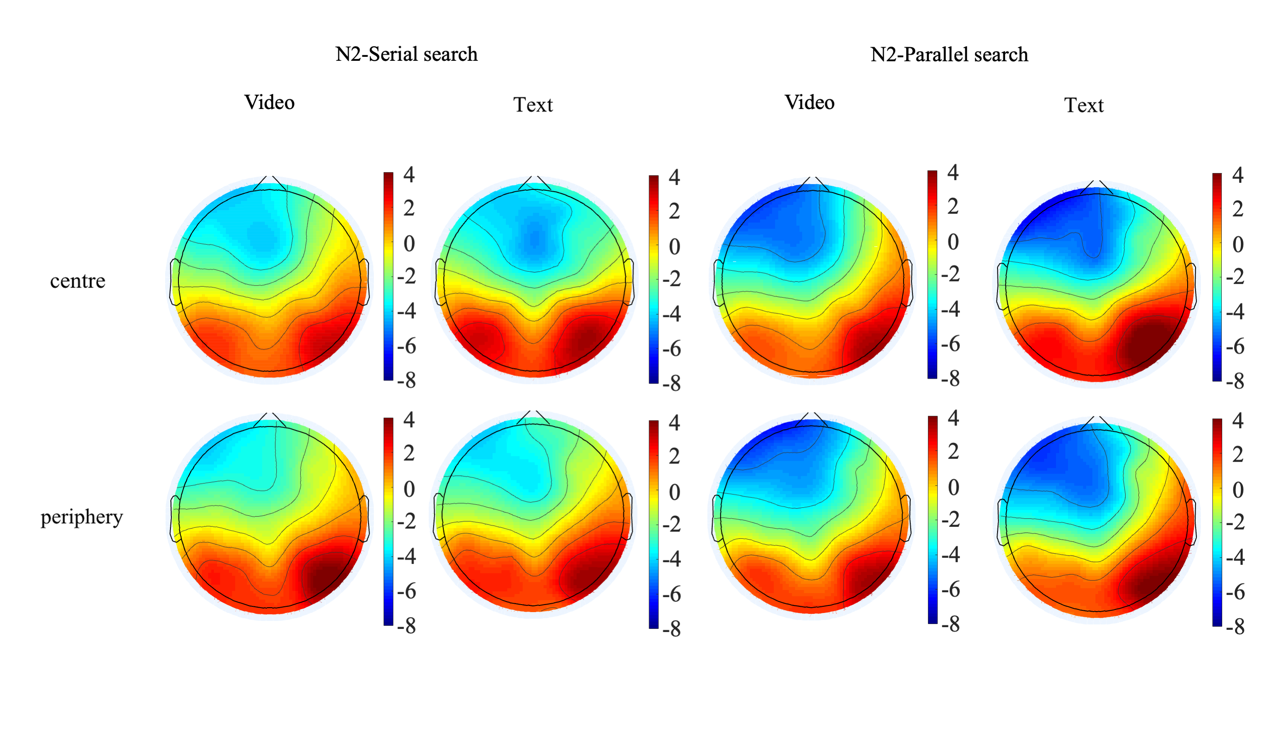

Supplement: Supplementary file 1 [file Data_Sheet_1.zip › Supplementary Materials/Figure 7.tif]

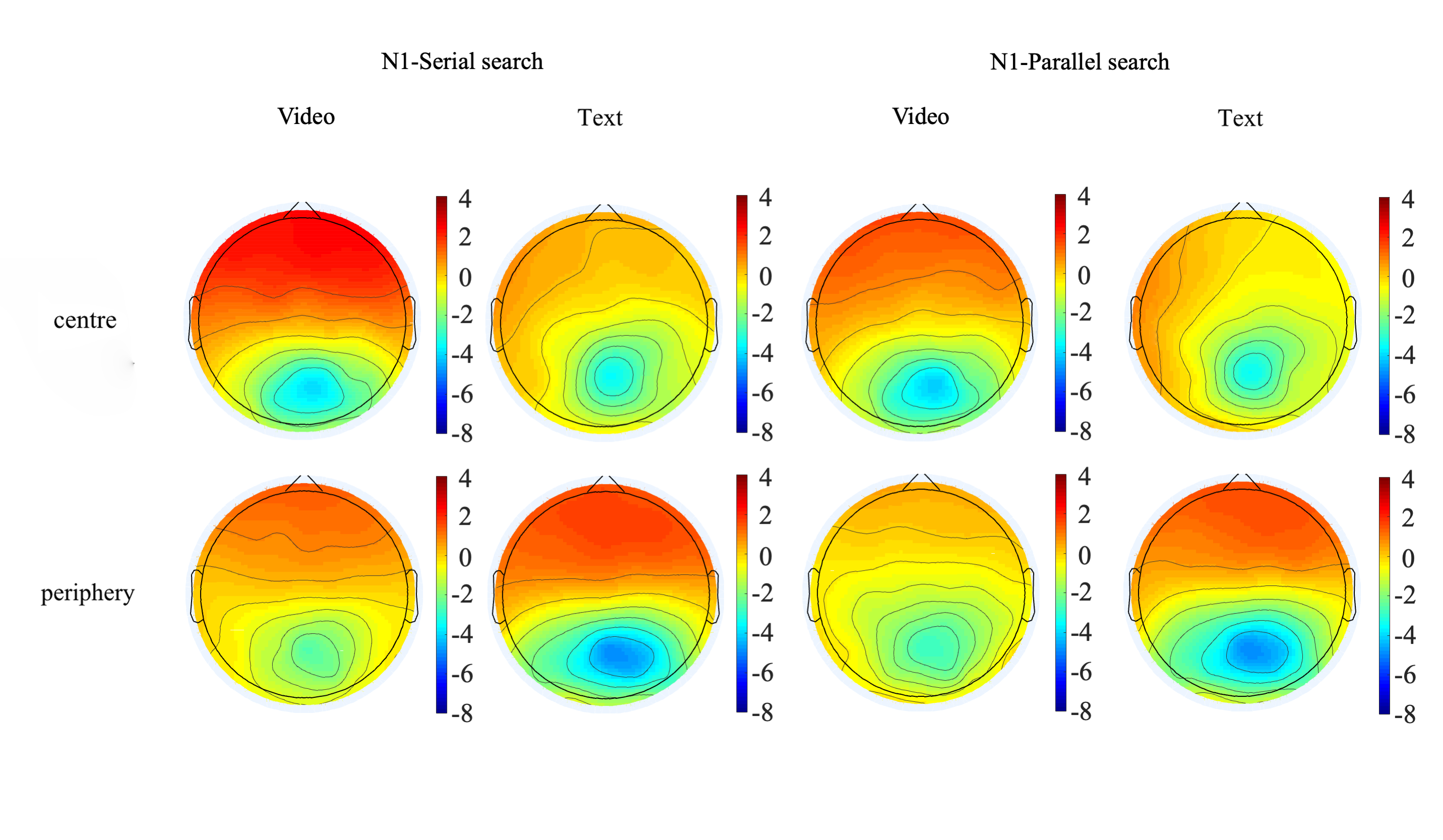

Supplement: Supplementary file 1 [file Data_Sheet_1.zip › Supplementary Materials/Figure 5.tif]

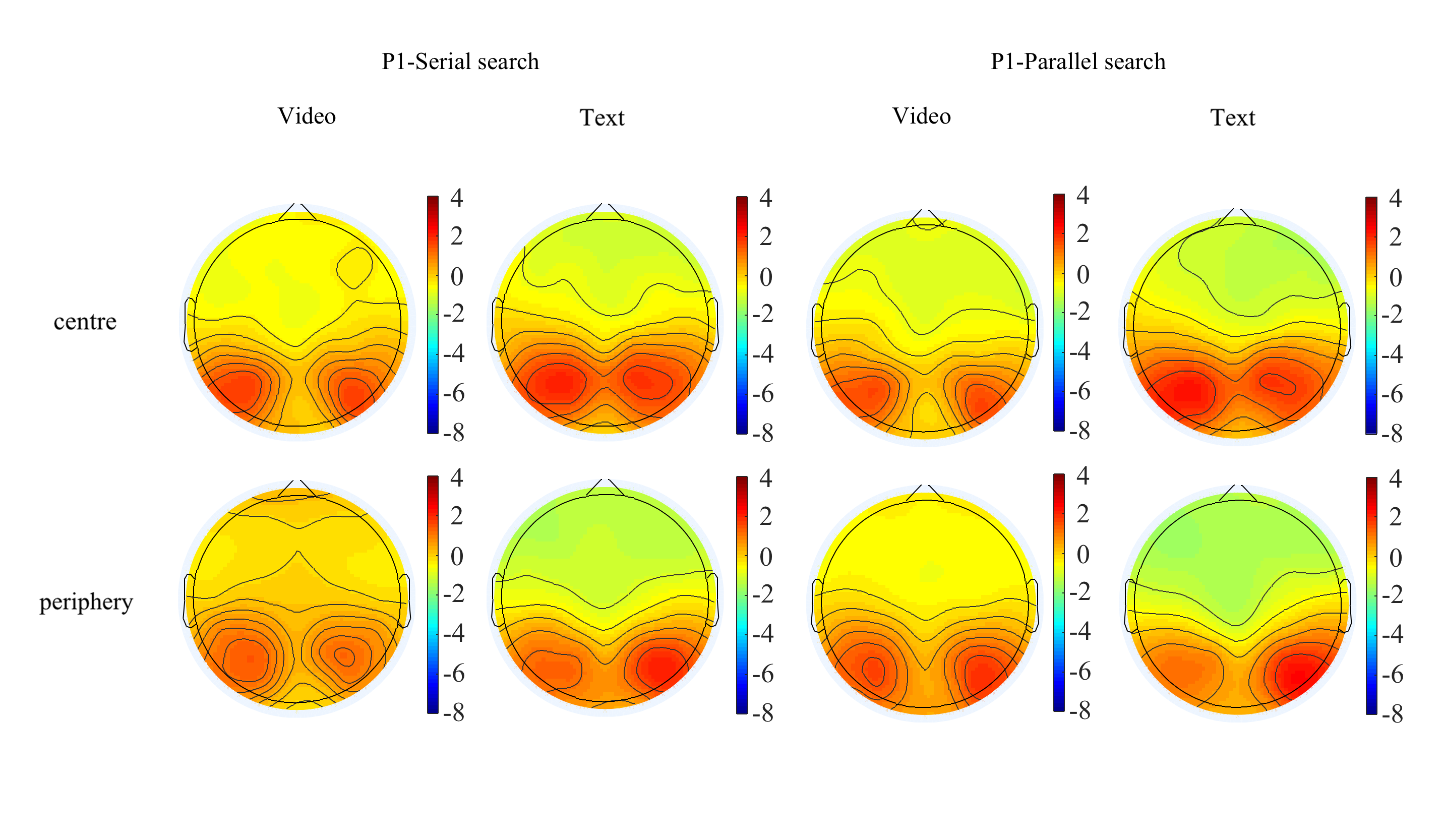

Supplement: Supplementary file 1 [file Data_Sheet_1.zip › Supplementary Materials/Figure 4.tif]

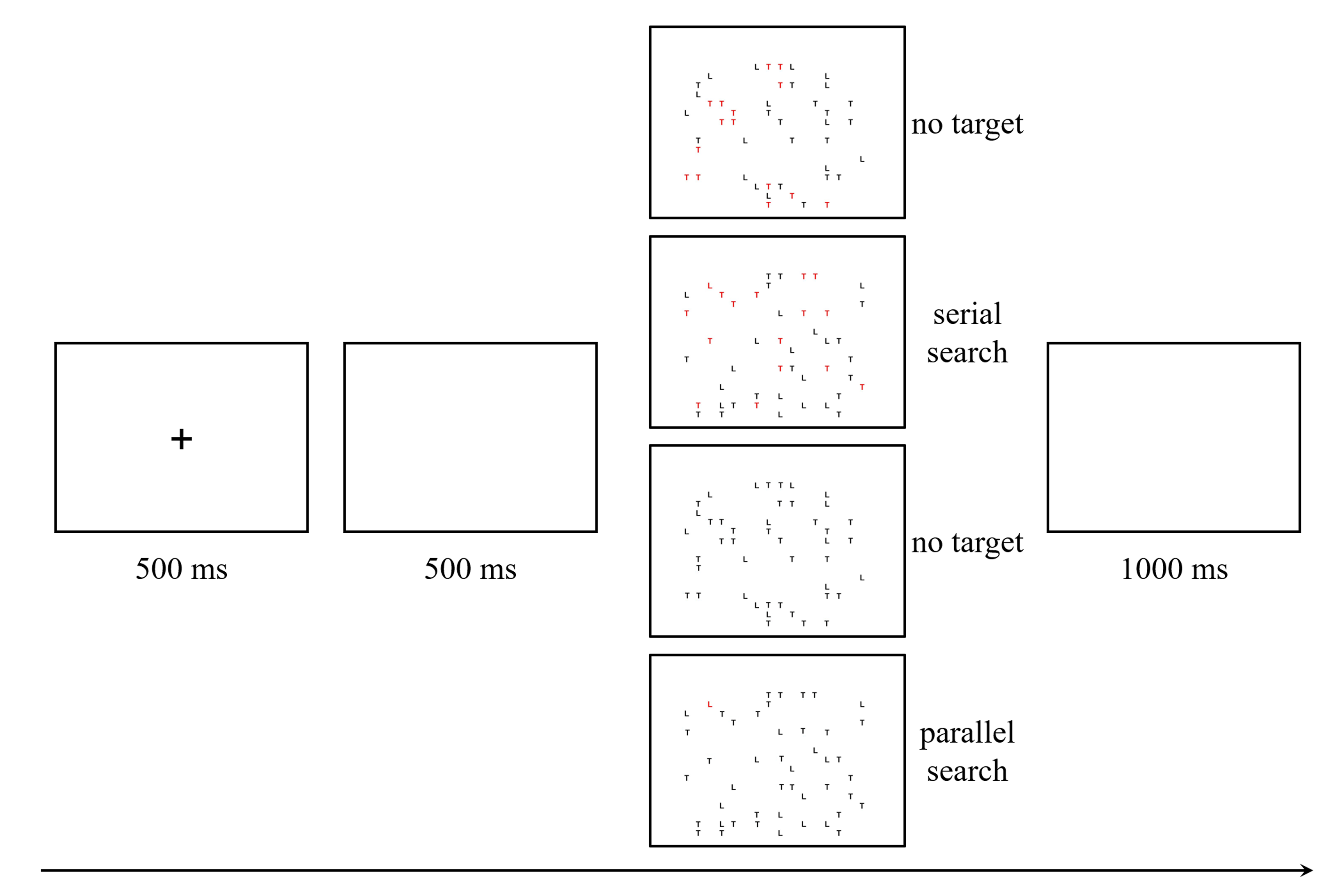

Supplement: Supplementary file 1 [file Data_Sheet_1.zip › Supplementary Materials/Figure 1.tif]

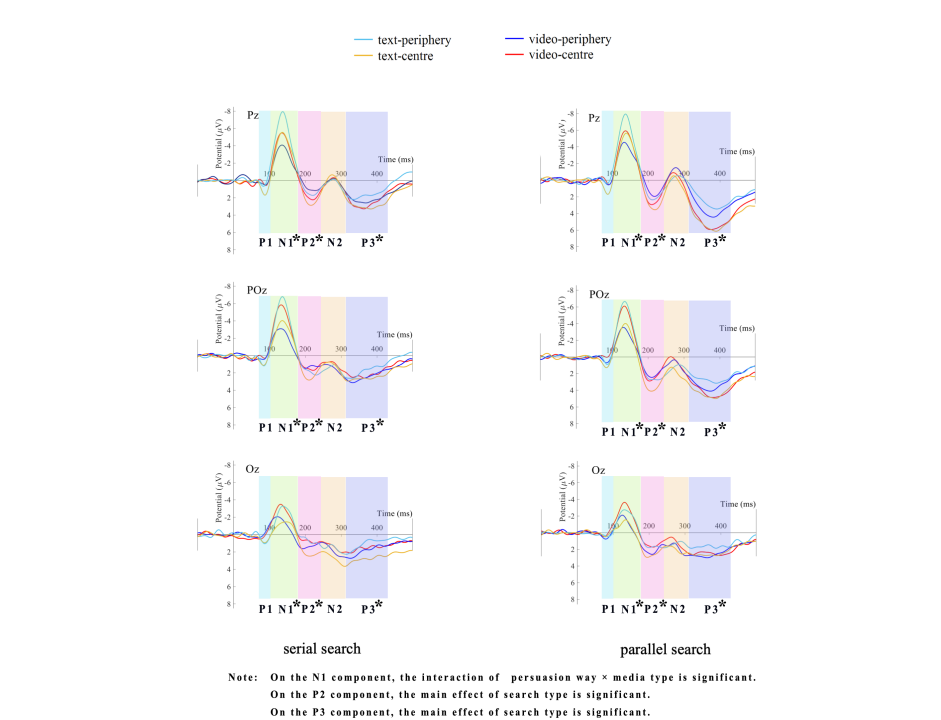

Supplement: Supplementary file 1 [file Data_Sheet_1.zip › Supplementary Materials/Figure 3.tif]

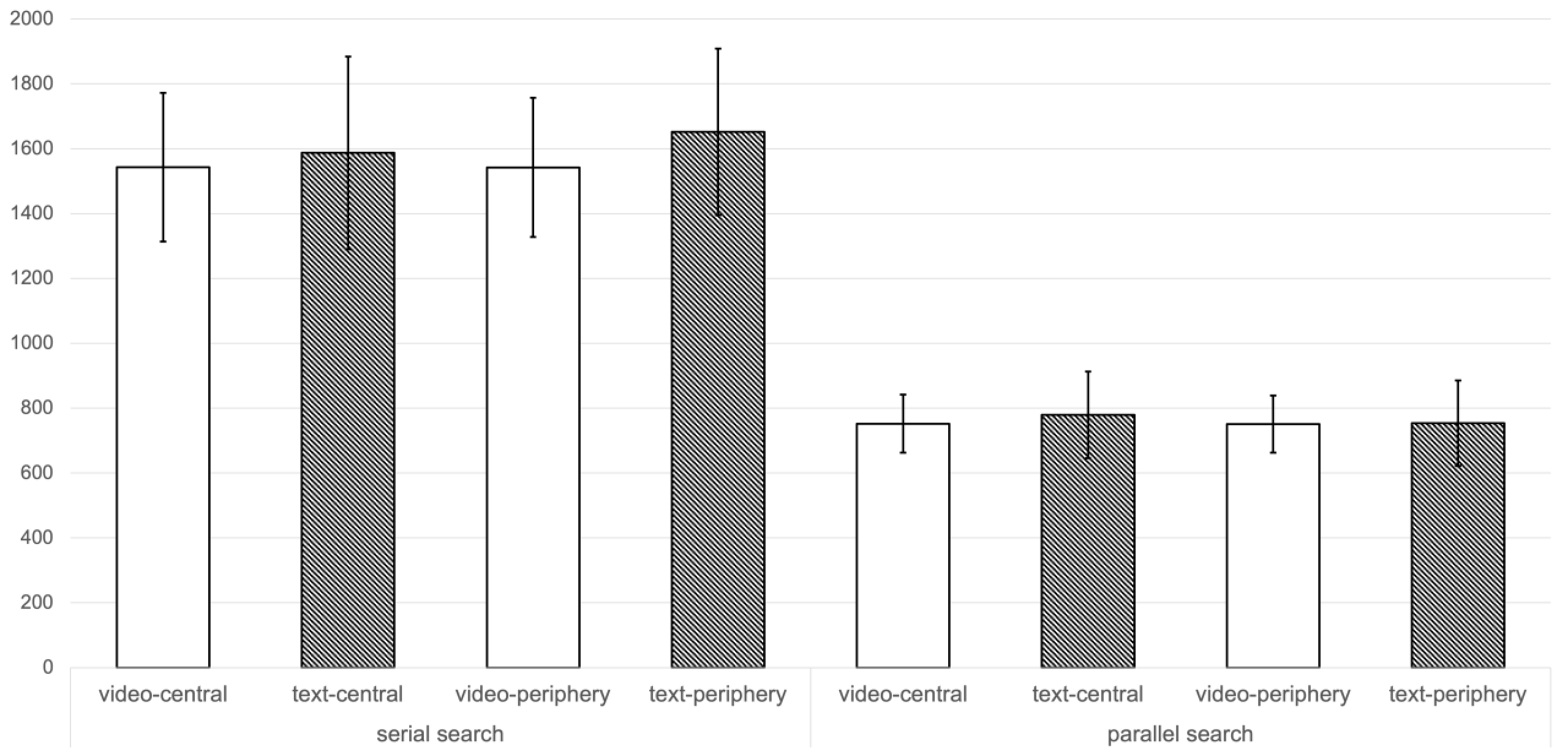

Supplement: Supplementary file 1 [file Data_Sheet_1.zip › Supplementary Materials/Figure 2.tif]

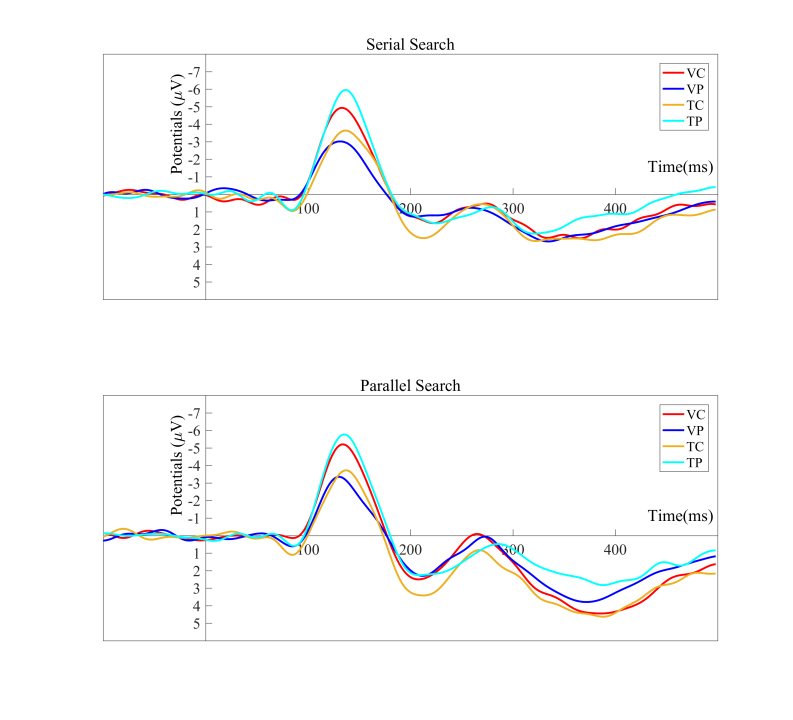

Supplement: Supplementary file 1 [file Data_Sheet_1.zip › Supplementary Materials/Figure 9.tif]

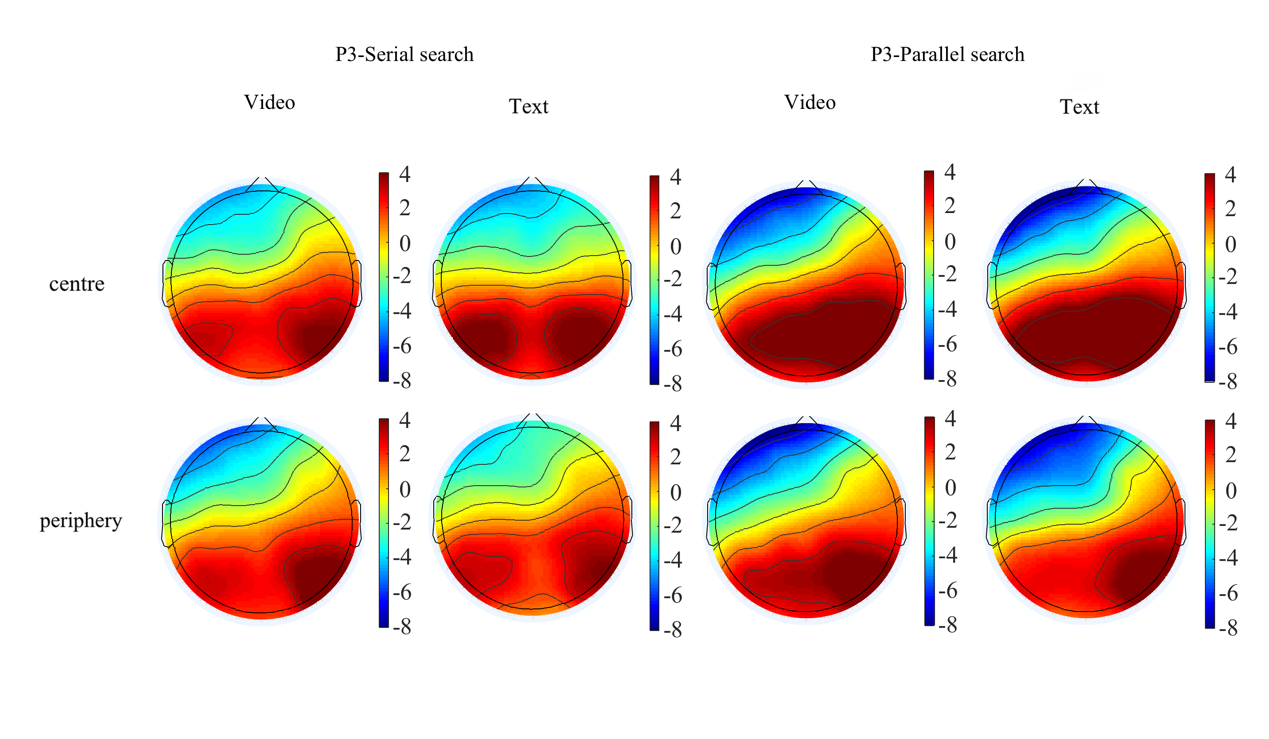

Supplement: Supplementary file 1 [file Data_Sheet_1.zip › Supplementary Materials/Figure 8.tif]
